# Effects of Biochar and Straw Amendment on Soil Fertility and Microbial Communities in Paddy Soils

**DOI:** 10.3390/plants13111478

**Published:** 2024-05-27

**Authors:** Hao Xia, Jia Shen, Muhammad Riaz, Cuncang Jiang, Chaolong Zu, Chaoqiang Jiang, Bo Liu

**Affiliations:** 1 Industrial Crop Institute, Anhui Academy of Agricultural Sciences (AAAS), Hefei 230001, China; 2 College of Resources and Environment, Zhongkai University of Agriculture and Engineering, Guangzhou 510225, China; 3 Microelement Research Center, College of Resources and Environment, Huazhong Agricultural University, Wuhan 430070, China; 4 Key Laboratory of Fertilization from Agricultural Wastes, Ministry of Agriculture and Rural Affairs, Institute of Plant Protection and Soil Fertilizer, Hubei Academy of Agricultural Sciences, Wuhan 430064, China

**Keywords:** biochar, straw, soil microorganism, soil DOM, carbon components

## Abstract

Straw and biochar, two commonly used soil amendments, have been shown to enhance soil fertility and the composition of microbial communities. To compare the effects of straw and biochar on soil fertility, particularly focusing on soil dissolved organic matter (DOM) components, and the physiochemical properties of soil and microbial communities, a combination of high-throughput sequencing and three-dimensional fluorescence mapping technology was employed. In our study, we set up four treatments, i.e., without biochar and straw (B0S0); biochar only (B1S0); straw returning only (B0S1); and biochar and straw (B1S1). Our results demonstrate that soil organic matter (SOM), available nitrogen (AN), and available potassium (AK) were increased by 34.71%, 22.96%, and 61.68%, respectively, under the B1S1 treatment compared to the B0S0 treatment. In addition, microbial carbon (MBC), dissolved organic carbon (DOC), and particulate organic carbon (POC) were significantly increased with the B1S1 treatment, by 55.13%, 15.59%, and 125.46%, respectively. The results also show an enhancement in microbial diversity, the composition of microbial communities, and the degree of soil humification with the application of biochar and straw. Moreover, by comparing the differences in soil fertility, DOM components, and other indicators under different treatments, the combined treatments of biochar and straw had a more significant positive impact on paddy soil fertility compared to biochar. In conclusion, our study revealed the combination of straw incorporation and biochar application has significant impacts and is considered an effective approach to improving soil fertility.

## 1. Introduction

Soils are a limited resource that sustain terrestrial life and can provide essential ecosystem functions for humans [1]. The maintenance and stability of soil fertility are crucial for ensuring food security in China [2]. However, intensive farming reduces organic matter, soil structure, and fertility [3]. Unreasonable fertilization harms soil quality, worsens pollution, and endangers human health [4]. For example, it leads to soil nutrient imbalances, structure destruction, and issues like acidification and salinization [5]. Additionally, the application of nitrogen, phosphorus, and potassium fertilizers can significantly reduce the richness of soil bacterial communities [6]. Thus, addressing how to improve the soil structure, farmland fertility, crop yield, and sustainable agricultural development is an urgent issue.

With the increase in agricultural production, the yield of crop straw has been consistently increasing and maintaining a high level. Returning crop straw to the field directly fertilizes the soil, improving its structure, fertility, and quality [7,8]. Straw contains rich nutrients (C, N, P, and K), which increase the content of soil organic carbon (SOC) and soil fertility [9]. Unreasonable straw returning to the field can also reduce crop emergence rates and exacerbate the occurrence of soil-borne diseases [10]. Additionally, biochar is a carbon-rich solid produced from agricultural and forestry waste through high-temperature and oxygen-limited conditions [11,12]. Biochar contains a considerable amount of organic carbon (>60%), and the carbon in biochar is mostly aromatic carbon [13,14]. Biochar can significantly reduce soil bulk density and increase the total soil porosity and moisture content [15,16]. Biochar, straw, and other organic materials have been shown to increase the soil organic carbon (SOC) content, improve soil structure, and enhance microbial community diversity [8]. However, the positive impact of biochar is particularly pronounced on acidic soils [12]. Biochar is primarily composed of inert carbon, whereas straw consists mostly of activated carbon that is more readily utilized by microorganisms [17]. Additionally, straw and biochar have different C/N ratios, leading to varying rates of decomposition. Straw decomposes organic matter like cellulose, polysaccharides, and proteins at a faster pace compared to biochar [18].

The soil carbon pool is crucial for soil quality and productivity, contributing significantly to its physical, chemical, and biological characteristics [19,20]. Unstable organic carbon is easily oxidized and decomposed, serving as an indicator for assessing soil quality and productivity [21]. Returning straw to the field can improve soil carbon storage and soil fertility in a short period of time [22]. To date, several studies have investigated how returning straw to the field can greatly enhance soil organic matter, humic acid, humin content, and humification [23,24]. A meta-analysis has shown that straw returning significantly increases soil organic carbon concentration and soil active carbon composition, promoting the formation of soil macroscopic aggregates [25]. Biochar application enhances soil organic carbon and microbial biomass carbon, thereby influencing soil carbon composition through improved microbial community structure [26,27]. Extracellular enzymes in the soil play an important role in regulating the decomposition of soil organic matter and nutrient cycling processes [28]. Various studies have assessed that biochar and straw can alter the activity of soil extracellular enzymes by affecting soil physicochemical properties [8,29].

In this study, we used straw and biochar as research materials to conduct field experiments on paddy soils in central China. Through field experiments, we investigated the impact of applying straw and biochar on soil nutrients, carbon components, enzyme activity, and the microbiome to reveal their potential for improving soil quality in paddy soils. We explored the connection between soil carbon composition, enzyme activity, and microbial community under various treatments using enzymatic techniques, soil DOM 3D fluorescence, and high-throughput sequencing. To be specific, the main hypotheses we wanted to validate in this study were the following: (1) explore the effects of straw and biochar on organic matter, carbon components, and DOM components in rice soil; (2) explore the intrinsic relationship between soil chemical factors and soil microbial communities; and (3) choose the most effective treatment to enhance the rice soil quality.

## 2. Materials and Methods

### 2.1. The Experimental Design

This field experiment was conducted at the Xiangyang experimental farm, Hubei province, China (112°07′19″ E, 32°00′36″ N). The study site is within the subtropical monsoon climate zone (15~16 °C), with precipitation and annual sunshine hours of 820~1100 mm and 1800~2100 h, respectively. The soil was paddy soil (Acrisols) collected from the topsoil layer (0–20 cm). The physicochemical properties of the tested soil were measured as described below. The organic matter was 24.51 g·kg^−1^; pH was 5.58; and the available N, P, and K were 92.77, 23.39, and 94.94 mg·kg^−1^, respectively. The straw used was wheat straw, with an average nutrient content of N (0.54%), P (0.09%), K (1.25%), and C (44.51%). The biochar was produced from rice straw at 500 °C, and its physical properties were analyzed as follows: pH, EC, C, N, P, and K were 9.60, 1850 µs/cm, 46.79%, 0.71%, 0.52%, and 5.30%, respectively. The specific fertilization information for each experiment is detailed as follows: the amounts of N, P, and K fertilizers applied in the rice season were 150, 39, and 75 kg/hm^2^, respectively; in the wheat season, the amounts of N, P, and K fertilizers applied were 120, 33, and 50 kg/hm^2^, respectively. Rice–wheat rotation is the typical cropping system in this area. Rice is typically transplanted in early June of each year and harvested in September, while wheat is usually sown in early October and harvested in early May of the following year. The trial commenced on 21 May 2019, with soil sampling conducted on 25 September 2021. Biochar, straw, and fertilizer were applied to the fields annually in May for a period of 3 years (2019–2021). The other field management practices remained consistent with the local farming practices.

The experiment consisted of four types of field management practices based on the results of our previous studies [8,12,29]: without biochar and straw (B0S0); biochar only (B1S0, 3.5 t/hm^2^); straw returning only (B0S1, 6 t/hm^2^); and biochar and straw (B1S1, 3.5 t/hm^2^ for biochar, 6 t/hm^2^ for straw), with four replicates of each treatment (n = 16). The size for each plot was 20 m^2^ (4 * 5 m). 

### 2.2. Sample Collection and Determination

The soil samples were collected from the tillage layer (0–20 cm), and all stones and large plant fragments were removed. The soil samples were divided into three parts: one part was air-dried and used to measure the soil basic chemical properties [30]; another part was stored at −20 °C and used to measure soil enzymatic activities; and another part was stored at −80 °C and used to measure soil microorganisms.

Soil pH and SOM were measured with a pH meter (water/soil  =  2.5/1) and chromic acid redox titration, respectively. Soil AK, AP, and AN were measured using the flame photometer method, molybdenum–antimony anti-spectrophotometric method, and alkali-hydrolytic diffusion method, respectively. MBC and MBN were measured using the chloroform fumigation–extraction method. DOC and DON were measured using a C/N analyzer. EOC and POC were measured using 333 mol L^−1^ of KMnO_4_ and (NaPO_3_)_6_, respectively. Fluorometry was used to measure the activities of soil extracellular enzymes (NAG, ACP, LAP, POX, and CAT) with a multifunctional microplate reader (Scientific Fluoroskan Ascent FL, Thermo, Waltham, MA, USA) [31].

The soil total DNA was extracted using the E.Z.N.A.^®^ DNA Kit (OmegaBio-tek, Norcross, GA, USA). DNA quality and purity were evaluated with a NanoDrop 2000 (NanoDrop Technologies, Wilmington, DE, USA) and visualized via electrophoresis on 1% agarose gels. The primers for bacteria were 515F (5′-GTGCCAGCMGCCGCGG-3′) and 806R (5′-GGACTACHVGGGTWTCTAAT-3′). The primers for fungi were ITS1F (5′-CTTGGTCATTTAGAGGAAGTAA-3′) and ITS2R (5′-GCTGCGTTCTTCATCGATGC-3′). PCR products were recovered and purified using 2% agarose gel and Axy Prep DNA Gel Extraction Kits(Axygen Biosciences, UnionCity, CA, USA). Sequencing was performed on an Illumina MiSeq with the assistance of Shanghai Majorbio Bio-pharm Technology Co., Ltd. 

Fluorescence Spectroscopy DOM: The soil sample was extracted with 1 mol·L^−1^ at 20 °C (200 r·min^−1^, 24 h). The samples were centrifuged (5000 r·min^−1^, 15 min) and filtered with a 0.45 µm glass fiber filter membrane [32]. The DOM solution was measured using a multi-mode spectroscopy analyzer (BioTek, Winooski, VT, USA). The excitation wavelength (λEx) was set at 5 nm, the emission wavelength (λEm) at 5 nm, and the scanning speed at 2400 nm/min. The measurement covered an excitation wavelength range of 200–440 nm and an emission wavelength range of 250–600 nm [33]. The C1, C2, and C3 represented different fluorescence component characteristics and were subjected to source analysis (Table S1).

### 2.3. Data Analysis

The sequencing of microorganisms was performed using the Fastp (version 0.20.0) and Flash software (version 1.2.7). We used the UPARSE software (version 7.0) to cluster OTU sequences and excluded chimeras with 97% similarity. The 16S rRNA database (Silva v138) and ITS database (Silva v138) were analyzed using a confidence threshold of 0.70. All sequencing data were combined for further analysis (alpha diversity analysis, beta diversity analysis, differential species analysis, and correlation and model prediction analysis) using the R software (version 4.2.3). Excel 2023 and SPSS Statistics 23.0 (IBM, Armonk, NY, USA) were used for data analysis. The data for the chemical properties of the soil are represented as the mean  ±  S.E. of triplicate data (n = 3). Fluorescence spectroscopy data were analyzed using MATLAB 2007. All graphs were created using the Origin software (version 8.0), R software (version 4.2), and Adobe Illustrator 2021.

## 3. Results

### 3.1. Effect of Various Treatments on Soil Chemical Properties in Paddy Soils

Table 1 shows that there are differences in the soil chemical properties under the different treatments. The SOM, AN, and AK differed significantly among the treatments, but the soil pH and AP did not show significant variations. Compared to the B0S0 treatment, the SOM, AN, and AK were increased by 34.71%, 22.96%, and 61.68%, respectively, under the B1S1 treatment (Table 1). Additionally, the AN and AK were increased by 22.28% and 46.48%, respectively, by the B0S1 treatment compared to the B1S0 treatment. These results show that biochar and straw addition could effectively increase soil fertility. The results were analyzed with a two-way ANOVA, and we found that straw had a significant influence on SOM, AN, and AK, while biochar only had a remarkable influence on SOM (*p* < 0.05) (Table 1).

A further analysis showed that the carbon (C) and nitrogen (N) components had different changes under the different treatments (Table 2). Compared to the B0S0 treatment, the MBC, DOC, and POC were significantly increased with the straw or biochar addition (Table 2). They increased from 174.67, 79.65, and 4.91 mg/kg under the B0S0 treatment to 270.97, 92.07, and 11.07 mg/kg under the B1S1 treatment (Table 2), respectively. The respective increases were 55.13%, 15.59%, and 125.46% in MBC, DOC, and POC. In addition, the DON also increased from 5.10 mg/kg under the B0S0 treatment to 6.46 mg/kg under the B1S1 treatment (Table 2). Furthermore, the MBC/MBN ratio increased with straw addition, while it decreased with biochar application (Table 2). Additionally, compared to the B1S0 treatment, the MBC, DON, and DOC were increased by 85.81%, 39.77%, and 34.06% under the B0S1 treatment (Table 2), respectively. Moreover, biochar had a significant influence on the soil MBC, DON, DOC, and POC, and straw also had noticeable effects on the soil MBC, DOC, and POC (*p* < 0.05) The interaction effect of biochar and straw on the soil MBC, DOC, and POC was significant (*p* < 0.05). The activities of ACP and NAG decreased with biochar or straw addition. Compared to the B0S0 treatment, the activities of ACP and NAG decreased by 14.42% and 11.69% under the B1S1 treatment (Table S2), respectively.

### 3.2. Effect of the Various Treatments on Three-Dimensional Fluorescence Spectral Characteristics of Soil DOM

A closer inspection of Table 3 reveals changes in the three-dimensional fluorescence spectral characteristics of soil DOM with biochar or straw addition. The FI decreased with biochar and straw addition (Table 3). Additionally, the BIX decreased by 11.29% under the B1S1 treatment compared to the B0S0 treatment, while the UV254 increased by 135.71% (Table 3). Moreover, Table 3 listed the proportions of C1, C2, and C3 as 46.32–49.28%, 44.34–44.43%, and 7.34–9.28%, respectively. Compared to the B0S0 treatment, C1 decreased from 49.28% to 46.41%, while C3 increased from 7.34% to 9.25% under the B1S1 treatment (Table 3).

### 3.3. Effect of the Various Treatments on Soil Bacteria and Fungi in Paddy Soils

**Figure 1:**
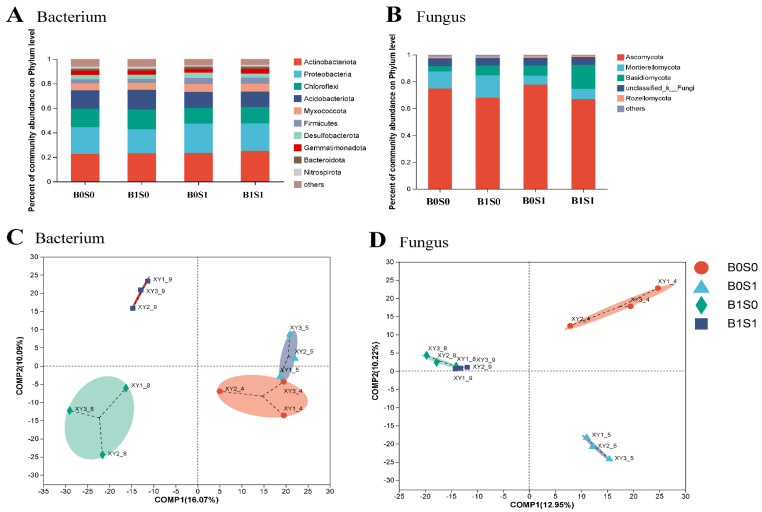
The community composition (**A**,**B**) and beta diversity (**C**,**D**) of soil bacterial (**A**,**C**) and fungal (**B**,**D**) communities under the different management measures.

**Figure 2:**
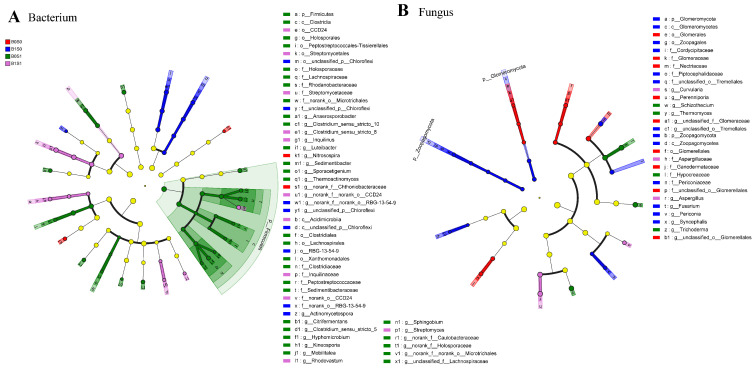
The LEfSe analysis of soil bacterial (**A**) and fungal (**B**) communities under the different management measures. The taxa with significantly different abundances among the different treatments are represented by dots with different colors, and from the center outward, they represent the phylum, class, order, family, and genus levels.

Soil samples were collected from various treatments to study the alterations in the soil microbial populations by biochar and straw application. The dominant bacteria were Actinobacteriota, Proteobacteria, Chloroflexi, Acidobacteriota, and Myxococcota (Figure 1A). Additionally, the dominant fungi were Ascomycota, Mortierellomycota, and Basidiomycota. Compared to the B0S0 treatment, the abundance of dominant genera significantly increased with biochar or straw addition (Figure 1B). We found a significant difference in the composition of microbial communities (bacteria and fungi) with biochar and straw addition (Figure 1C,D). Among all treatments, the number of dominant genera was the highest under the B0S1 treatment, while it was the lowest under the B0S0 treatment (Figure 2A and Figure S1A). The top five biomarkers were p_Firmicutes, c_Clostridia, o_Xanthomonadales, f_Rhodanobacteraceae, and f_Clostridiaceae with straw addition alone. However, g_norank_f_Chthoniobacteraceae and g_Nitrosospira were enriched under the B0S0 treatment (Figure 2B and Figure S1B). Unlike bacterial changes, the number of dominant genera in fungi was the highest with only biochar addition, while it was the highest under the B1S1 treatment. The top five biomarkers were g_Fusarium, f_Cordycipitaceae, g_unclassified_o_Tremellales, f_unclassified_o_Tremellales, and p_Glomeromycota under the B1S0 treatment, while g_Aspergillus, f_Aspergillaceae, and g_Curvularia were enriched with the B1S1 treatment (Figure S1B).

### 3.4. The Relationship between Soil Environment and Microorganisms under Different Treatments

**Figure 3:**
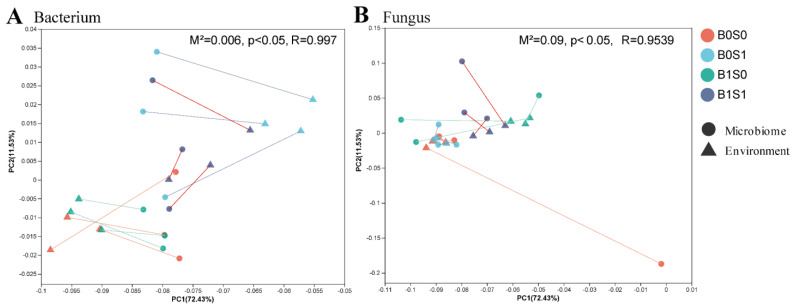
The Procrustes analysis between the soil microbiome (soil bacteria (**A**) and fungi (**B**)) and environment index under the different management measures.

**Figure 4:**
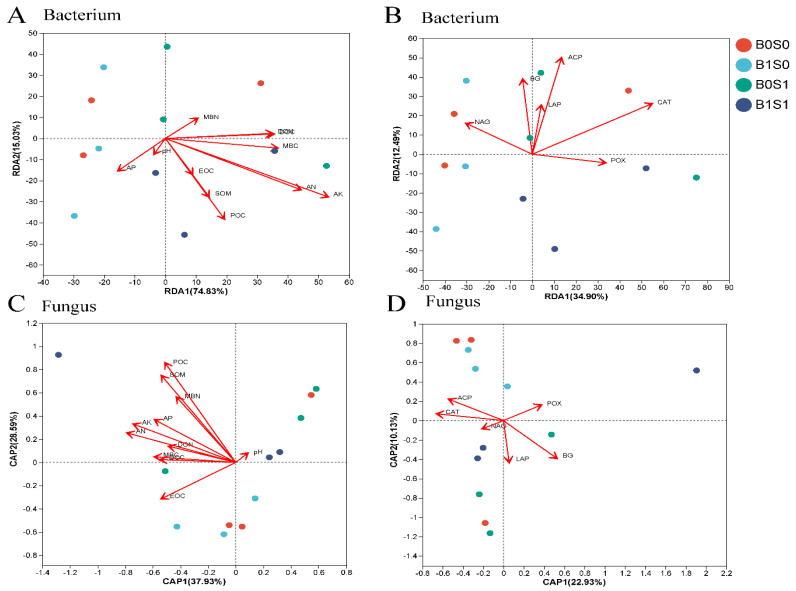
Redundancy analysis (RDA) based on the OTU level between the soil microbiome (soil bacteria (**A**,**B**) and fungi (**C**,**D**)) and environment index (soil fertility indicators (**A**,**C**) and soil enzyme activity (**B**,**D**)) under the different management measures.

**Figure 5:**
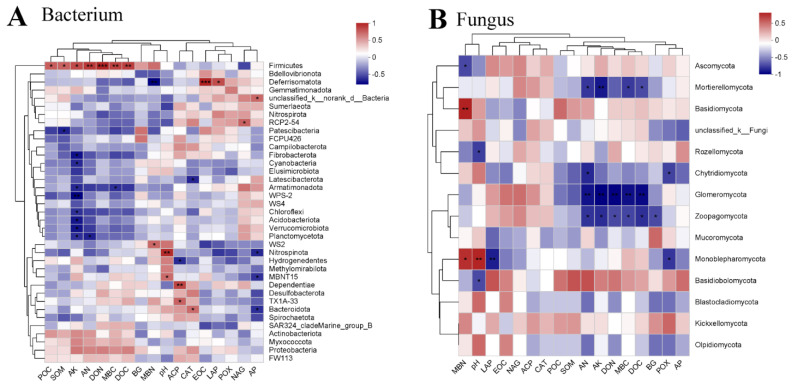
The correlation analysis between the soil microbiome (soil bacteria (**A**) and fungi (**B**)) and environment index under the different management measures. * Indicates correlation at different levels (* *p* < 0.05, ** *p* < 0.01, *** *p* < 0.001).

Our subsequent objective was to investigate the connections between the soil microbiome and environmental factors. It was found that there was a tight link between the soil microbiome (bacteria and fungi) and environmental factors (*p* < 0.05) (Figure 3). The Procrustes analysis findings (bacteria R = 0.9970, *p* < 0.05; fungi R = 0.9539, *p* < 0.05) show that the soil bacteria and fungi varied significantly with the soil chemical properties (Figure 3). Additionally, the analysis of the RDA indicated that the soil microbiome (bacteria and fungi) changed in response to the surrounding conditions (Figure 4). AK, AN, POC, and MBC were the main important environmental factors for soil bacterial community composition, and ACP, βG, and CAT were important soil enzymes for soil bacteria (Figure 4A,B). Moreover, POC, SOM, AN, AK, and MBN were major soil environmental indices for soil fungi, and ACP, CAT, POX, and *β*G were also prominent soil factors for the fungal community structure (Figure 4C,D). In addition, a correlation analysis was performed using a Pearson correlation analysis to explore the relationship between the soil microbiome (bacteria and fungi) and environmental factors (Figure 5). The results in Figure 5A also demonstrate that AK is the most important index influencing the soil bacterial community composition, and most soil bacterial communities always maintain significant (*p* < 0.05) negative associations with the soil AK. The relative abundance of Firmicutes had a strong positive association with the soil chemical properties (Figure 5A). Moreover, the soil AN, AK, DON, MBC, and DOC had significant negative correlations with soil fungi at the phylum level (Mortierellomycota, Glomeromycota, and Zoopagomycota) (Figure 5B).

## 4. Discussion

### 4.1. Effects of Biochar and Straw on the Soil Chemical Properties in Paddy Soils

Soil nutrient levels impact plant growth, metabolism, and other factors [34]. The current study found that biochar and straw significantly improved soil available nutrients, especially carbon components (Table 1 and Table 2). There are several possible explanations for this result: (1): Biochar and straw are organic materials rich in carbon, which can increase soil organic carbon concentration and improve soil nutrient status [35,36]. (2): Returning biochar and straw to the field boosts soil microbial activity, leading to increased nutrient release and improved soil organic matter and fertility [8]. (3): Biochar and straw can improve crop root biomass, enhancing soil organic carbon and nutrient levels [37]. The study found that the AN did not significantly change with varying amounts of biochar application (Table 1). This could be attributed to the fact that an excessive biochar (high C/N ratio of biochar) application may enhance microbial nitrogen fixation, leading to a reduced nitrogen availability for plants in the soil and lower nitrogen mineralization utilization rate [38]. Xia et al. [39] found crop straw and biochar returning to the soil significantly increased the soil carbon component (DOC, MBC, POC, and ROC). Another important finding was that biochar had more distinct effects on the soil carbon components (Table 2). Several factors could explain this observation. (1): Biochar contains rich pore structures, which can reduce the rate of soil nutrient release and reduce nutrient loss [40]. (2): Biochar has a higher charge density, which can effectively absorb soil nutrients and improve nutrient utilization [41]. (3): Biochar is more decomposed, and soluble carbon is more readily available under pyrolysis treatment at high temperatures [42]. (4): The carbon in straw is mainly activated carbon, while the carbon in biochar is mostly inert [43]. One interesting finding was that some available nutrients were increased less notably compared to the biochar treatment with straw addition (Table 1). A possible explanation for this might be that the chemical properties and pore structure are changed in straw during the pyrolysis process [44]. The content of elements (N, H, and O) in biochar decreases with the increase in the carbonization temperature [45]. The strong adsorption of biochar in soil pores can reduce the availability of free nutrients [46]. Many reports found that soil enzymes were affected by different management measures [47].

Extracellular enzymes in the soil participate in soil biogeochemical cycling through processes such as the catalysis, degradation, transformation, and synthesis of soil organic matter [48]. The results of this study indicate that the soil extracellular enzymes (ACP and NAG) changed with biochar and straw addition (Table 3). The activities of the soil βG, CBH, NAG, and LAP significantly increased with straw and biochar addition [8]. A possible explanation for this might be that straw and biochar could provide soil available nutrients for soil microorganisms, which could incite soil enzymes [49]. Moreover, the soil enzyme activity had varying responses to biochar and straw addition in our study (Table 3). This discrepancy could be attributed to two factors: (1) Biochar pores can adsorb necessary substrates, boost soil enzyme activity, and enhance enzymatic reaction efficiency. (2) The potentially harmful substances (organic pollutants and heavy metals) in biochar decrease soil enzymes and microbial activity [50]. Compared to the application of biochar alone, straw application is more effective in enhancing soil fertility and increasing carbon components (Table 1 and Table 2). We hypothesize that this may be due to one of the following reasons: As the carbonization temperature increases during the biochar preparation process, the content of active carbon components in the biochar decreases [51]. (1) Substances with a high carbon-to-nitrogen (C/N) ratio are less likely to decompose in soil. Thus, the carbon present in straw is more readily accessible to soil microorganisms compared to the carbon in biochar [52]. (2) Straw application can lead to an excitation effect on soil organic matter in the initial stages, while biochar tends to remain stable in the soil as it is resistant to decomposition [53].

### 4.2. Effects of Biochar and Straw on Soil 3D Fluorescence Spectral Characteristics of Soil DOM in Paddy Soils

Soil dissolved organic matter (DOM) is an active component of SOM, composed of humic acids, proteins, organic acids, and amino acids [54]. Dissolved organic matter (DOM) plays a critical role in the breakdown of soil nutrients, as well as in the growth and metabolism of microorganisms in the field [55]. The current study found that the value of FI was greater than 1.9 under all treatments, suggesting that microbial metabolism provides the majority of DOM [56]. Moreover, the value of the BIX lower than 1 under all treatments indicated the autochthonous or biological production of DOM components sourced from terrestrial DOM [57]. In addition, the FI decreased with the biochar and straw treatment (Table 3). These results suggest biochar and straw increase the soil microbial diversity, promoting the decomposition of plant residues [58]. Biochar exhibits a strong chemical stability and has the ability to absorb soil organic molecules as it undergoes a slow decomposition process, ultimately aiding in the formation of humus [59]. Furthermore, the value of UVA254 increased with biochar and straw addition, indicating the degree of soil SOM humification was enhanced with straw and biochar addition (Table 3). This result may be explained by the fact that returning straw to the field is beneficial for increasing the degree of DOM humification and the hydrophobic component content [32]. Moreover, straw and biochar promoted the percentage of C3, while decreasing the percentage of C1 (Table 3). Straw and biochar decomposition increases soil DOM aromaticity, humification degree, and molecular weight, leading to the accumulation of tyrosine, humic acid, and fulvic acid components [60]. Wu et al. [61] found microorganisms break down straw in the field to convert organic carbon into smaller DOM fractions. However, some studies have demonstrated that the porous structure of biochar readily adsorbs soluble organic carbon from the soil, leading to an encapsulation effect and suppression of soil microorganism activity, ultimately resulting in a reduction in soil organic carbon mineralization [62]. In addition, the return of straw to fields has a limited impact on increasing soil organic carbon storage in the short term, with carbon emissions during its rapid decomposition being the primary source of greenhouse gases, leading to negative environmental consequences [63].

### 4.3. Effect of Various Treatments on Soil Microorganisms in Paddy Soils

In our study, we also found that biochar and straw alter the composition of the soil microbial community (Figure 1). There are multiple potential explanations for this result [8,12,46]: (1) straw and biochar may offer readily available nutrient content and carbon sources for soil microorganisms; (2) the combination of biochar and straw could establish optimal soil moisture and temperature conditions, thereby enhancing the diversity of soil microbial communities; and (3) biochar and straw may serve as effective shelters for microorganisms. In addition, straw and biochar could increase the abundance of beneficial microorganisms in paddy soils (Figure 2 and Figure S2). For example, straw could promote the abundance of p_Firmicutes, and biochar could promote the abundance of p_Glomeromycota and p_Zoopagomycota (Figure S2). Several factors could explain this observation: (1) adding organic matter can supply carbon sources and energy, which greatly increases soil microbial activity (particularly the growth of R-type microorganisms) [64]; and (2) adding different organic substances to the soil to keep a variety of organic matter for soil microorganisms, maintaining diversity in agricultural ecosystems [65]. Zhou et al. [66] found that adding more than six types of organic matter can effectively prevent tomato wilt disease (particularly in carbohydrates and fatty acids). Based on the plant–soil feedback effect, long-term intensive agriculture leads to a decrease in the soil organic matter content, nutrient depletion, and the frequent occurrence of soil-borne diseases [67]. In our study, biochar and straw are common organic materials that can greatly enhance soil organic matter and available nutrients (Table 1 and Table 2). These findings could be used to explain why exogenous organic matter can alter the structure of soil microbial communities. In addition, substances such as polycyclic aromatic hydrocarbons (PAHs), volatile organic compounds (VOCs), and environmental persistent free radicals (EPFRs) found on the surface of biochar have the potential to impede the growth and metabolic functions of specific microorganisms [68]. Furthermore, through a correlation analysis, Procrustes analysis, and redundancy analysis, there was a close relationship between soil microorganisms and soil physicochemical properties (Figure 3, Figure 4 and Figure 5). For example, the relative abundance of p_Firmicutes was significantly correlated with the soil nutrients (POC, SOM, AK, AN, DON, and MBC). Many studies found that p_Firmicutes were crucial for facilitating plant nitrogen fixation, organic matter degradation, environmental remediation, soil fertility, and crop growth within the ecological environment [69,70]. 

Microorganisms can decompose organic matter, promote crop growth, and maintain the nutritional balance and health of the soil [71,72]. Soil pH changes can impact nitrogen-fixing bacteria abundance and community structure, reduce nitrification-related microorganisms (p_Nitrospinota), and inhibit nitrifying bacteria activity [73,74]. Gao et al. [75] found that soil bacterial and fungal communities increased with more biochar, and the timing of biochar application affected soil microbial diversity. In addition, a negative correlation was found between the relative abundance of soil fungi (p_Glomeromycota and p_Zoopagomycota) and soil nutrient level (Figure 5). The p_Glomeromycota can form arbuscular mycorrhizas with terrestrial plants, which can assist plants in absorbing inorganic salts and nutrients from the soil, especially in poor soils [76,77]. Thus, applying straw or biochar to the soil can activate soil microorganisms, maintain soil microbial diversity, and improve soil health.

## 5. Conclusions

This study investigates the impact of straw and biochar on soil fertility, organic matter, dissolved organic matter (DOM) components, and microbial communities in field experiments. The findings indicate that biochar and straw effectively enhanced soil fertility, particularly soil organic matter and available potassium levels. In addition, biochar and straw increased soil particulate organic carbon (POC), microbial biomass carbon (MBC), and total organic carbon contents to different extents. When compared, straw has a more pronounced effect on increasing the soil carbon component than biochar. Furthermore, biochar and straw increased the soil microbial diversity, which promoted the decomposition of plant residues. Additionally, the degree of soil SOM humification was enhanced with straw and biochar addition. Thus, biochar and straw enhanced the structure of microbial communities and suppressed the proliferation of harmful bacteria through the restoration of soil organic matter and essential nutrients. In general, the utilization of biochar, straw incorporation into the soil, and the combination of straw incorporation and biochar application have been shown to enhance soil fertility, soil carbon sequestration capacity, and the soil microenvironment. Particularly, the combination of straw incorporation and biochar application has demonstrated the most significant impact and is considered an effective approach to improving soil fertility.

## Figures and Tables

**Table 1:** Effects of biochar and straw on soil basic chemical properties in paddy soils.

Treatment	pH	SOM (g/kg)	AN (mg/kg)	AP (mg/kg)	AK (mg/kg)
B0S0	5.61 ± 0.12 a	24.66 ± 0.67 c	97.86 ± 7.08 b	29.05 ± 3.02 a	91.04 ± 4.72 b
B1S0	5.76 ± 0.17 a	29.47 ± 2.13 b	92.81 ± 7.12 b	30.11 ± 3.85 a	99.36 ± 6.48 b
B0S1	5.65 ± 0.23 a	30.92 ± 2.22 ab	113.49 ± 5.83 a	30.68 ± 12.07 a	145.54 ± 20.62 a
B1S1	5.61 ± 0.15 a	33.22 ± 2.03 a	120.23 ± 2.2 a	34.24 ± 3.87 a	147.2 ± 10.47 a
B	0.28	10.78 *	0.06	0.35	0.50
S	0.32	21.43 **	39.80 **	0.54	52.44 **
B*S	0.86	1.35	2.98	0.10	0.22

Note: Lowercase letters indicate significant differences in biochar and straw addition among the different treatments with a Duncan’s test (*p* < 0.05). Values are the means ± SD (n = 3). The statistical results of a two-way analysis of variance are expressed as F-value and *p*-value. * *p* < 0.05; ** *p* < 0.01.

**Table 2:** Effects of biochar and straw additions on soil carbon and nitrogen components in paddy soils.

Treatment	MBN (mg·kg^−1^)	MBC (mg·kg^−1^)	DON (mg·kg^−1^)	DOC (mg·kg^−1^)	EOC (mg·kg^−1^)	POC (mg·kg^−1^)	MBC/MBN	DOC/DON
B0S0	4.59 ± 0.67 a	174.67 ± 24.57 c	5.10 ± 0.60 b	79.65 ± 3.85 c	21.91 ± 2.13 a	4.91 ± 0.27 c	38.11 ± 2.90 ab	15.71 ± 1.26 a
B1S0	5.81 ± 0.32 a	190.75 ± 5.43 c	5.13 ± 0.48 b	81.12 ± 1.16 c	21.08 ± 2.56 a	8.20 ± 1.43 b	32.95 ± 2.83 b	15.92 ± 1.64 a
B0S1	6.75 ± 1.80 a	354.43 ± 13.00 a	7.17 ± 0.50 a	108.75 ± 2.79 a	19.79 ± 1.94 a	7.71 ± 1.25 b	55.39 ± 16.07 a	15.23 ± 1.36 a
B1S1	6.28 ± 1.53 a	270.97 ± 36.37 b	6.46 ± 0.08 a	92.07 ± 6.86 b	22.98 ± 2.19 a	11.07 ± 1.33 a	43.98 ± 6.02 ab	14.27 ± 1.15 a
B	3.37	95.41 **	41.18 **	67.88 **	0.36	0.01 *	7.73 *	1.85
S	0.27	6.41 *	1.67	9.78 *	0.01	0.86 *	2.65	0.23
B*S	1.39	13.99 **	1.96	13.93 **	1.03	2.47 *	0.38	0.56

Note: Lowercase letters indicate significant differences in biochar and straw addition among the different treatments with a Duncan’s test (*p* < 0.05). Values are means ± SD (n = 3). The statistical results of two-way analysis of variance are expressed as F-value and *p*-value. * *p* < 0.05; ** *p* < 0.01.

**Table 3:** Effects of biochar and straw addition on soil DOM fluorescence components, fluorescence spectral parameters, and ultraviolet spectral parameters in paddy soils.

Treatment	Fluorescence Index (FI)	Biological Index (BIX)	UV254	The Distribution of Fluorescence Components		
				C1%	C2%	C3%
B0S0	2.10 ± 0.03 a	0.62 ± 0.04 a	0.14 ± 0.02 c	49.28 ± 1.48 a	43.38 ± 2.47 a	7.34 ± 0.99 a
B1 S0	1.98 ± 0.03 b	0.55 ± 0.02 a	0.26 ± 0.01 b	49.72 ± 2.46 a	44.43 ± 6.22 a	9.18 ± 1.85 a
B0S1	2.06 ± 0.05 ab	0.57 ± 0.08 a	0.23 ± 0.02 b	46.32 ± 8.66 a	44.41 ± 7.47 a	9.28 ± 1.20 a
B1S1	2.02 ± 0.09 ab	0.55 ± 0.04 a	0.33 ± 0.01 a	46.41 ± 4.37 a	44.34 ± 2.58 a	9.25 ± 2.61 a
B	5.83 *	1.81	124.12	0.01	0.03	0.78
S	0.01	0.68	71.79 **	1.15	0.02	0.95
B*S	1.24	0.89	1.19	0	0.03	0.83

Note: Lowercase letters indicate significant differences in biochar and straw addition among the different treatments with a Duncan’s test (*p* < 0.05). Values are means ± SD (n = 3). The statistical results of two-way analysis of variance are expressed as F-value and *p*-value. * *p* < 0.05; ** *p* < 0.01.

## Data Availability

Data are contained within the article and Supplementary Materials.

## Supplementary Materials

The following supporting information can be downloaded at: https://www.mdpi.com/article/10.3390/plants13111478/s1. Figure S1: The three fluorescence fractions and loading maps of DOM of paddy soil. (A–C): represented the different fluorescence fractions; (D): Different Fluorescence characteristics and source analysis; Figure S2: The LEfSe analysis of soil bacterial (A) and fungus (B) under different management measures. The taxa with significantly different abundances among different treatments are represented by dots with different colors, and from the center outward, they represent the phylum, class, order, family, and genus levels; Table S1: Description and significance of characteristic parameters of ultraviolet-visible spectroscopy of dissolved organic matter; Table S2: Effects of biochar and straw on soil enzyme activity in paddy soil.
