# Supplementary material for: Effects of Biochar and Straw Amendment on Soil Fertility and Microbial Communities in Paddy Soils
Source: Plants (Basel). 2024 May 27;13(11):1478. doi: 10.3390/plants13111478 (PMC11174402; doi:10.3390/plants13111478)
Supplement: Supplementary file 1 [file plants-13-01478-s001.zip › plants-3004942-supplementary.pdf]

Table S1 Description and significance of characteristic parameters of ultraviolet-visible spectroscopy of dissolved organic matter

Index	Characterization
Fluorescence index (FI)	Characterization of Humus Sources in DOM
Humification index (HI)	Characterizing the degree of humification of DOM
Biological index (BI)	Characterizing the strength of DOM autogenous source features
UV254 nm	Characterizing the degree of DOM humification

Table S2 Effects of biochar and straw on soil enzyme activity in paddy soil.

Treatment	ACP (ug.h^{-1}. g^{-1})	NAG (ug.h^{-1}. g^{-1})	LAP (ug.h^{-1}. g^{-1})	POX (ug.h^{-1}. g^{-1})	CAT (ug.h^{-1}. g^{-1})
B0S0	97.61±14.89a	105.33±7.55a	104.11±9.96a	0.82±0.22ab	7.98±0.93a
B1S0	88.63±11.40a	106.98±12.35a	102.39±11.65a	0.62±0.25ab	8.56±0.76a
B0S1	86.99±8.46a	91.29±20.05a	94.22±16.19a	0.58±0.05b	8.20±0.97a
B1S1	83.53±8.35a	93.02±8.99a	108.98±12.97a	0.99±0.20a	7.71±0.98a
B	1.51	0.29	3.39	0.05	0.34
S	0.94	1.38	0.05	0.77	0.88
B*S	0.19	1.53	0.00	1.23	7.09

Note: Lower case letters indicated significant differences in biochar and straw addition among different treatments with a Duncan test ($P < 0.05$). Values are means ± SD (n = 3). The statistical results of two-way analysis of variance are expressed in F-value and P-value. *$P < 0.05$; **$P < 0.01$.

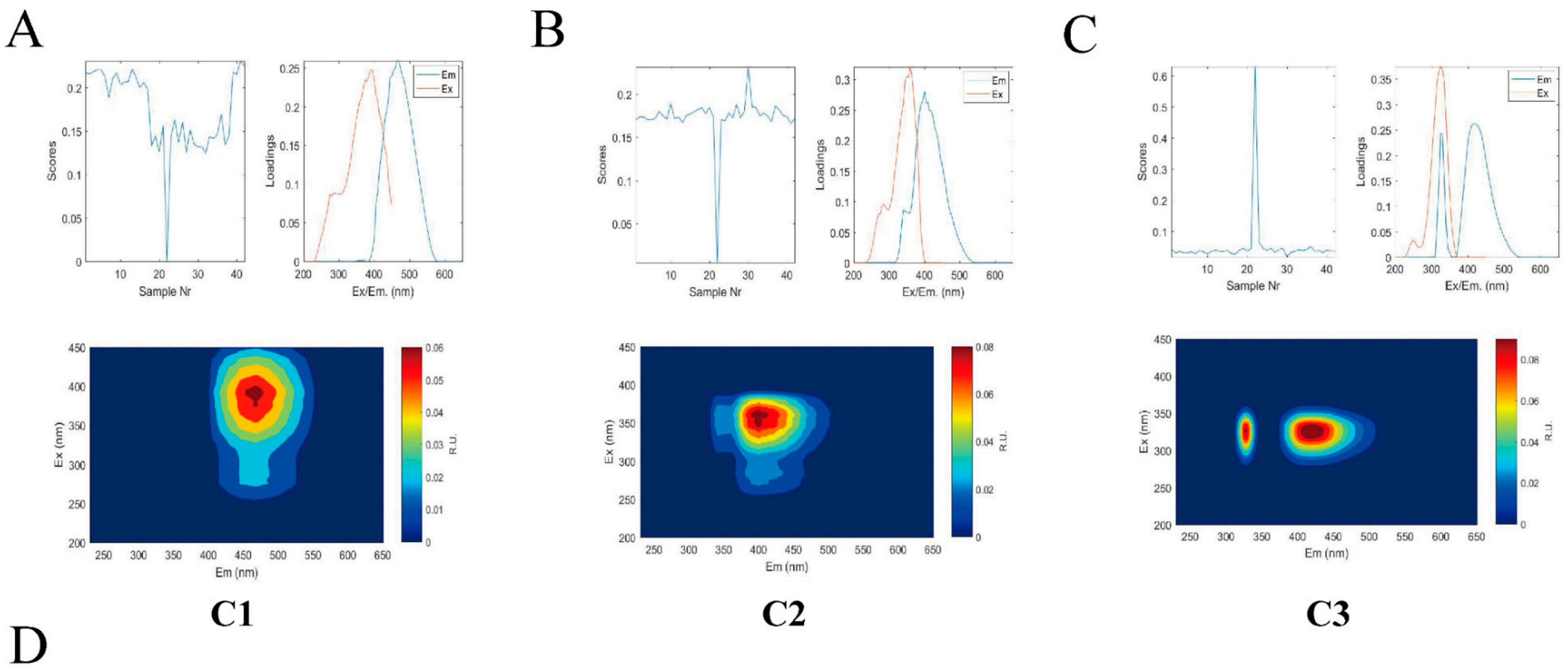

D

Component	Ex/ Em/nm	Type
C1	EX390/Em460	Humus like substances, Reducing semiquinones
C2	EX360/Em400	Humus like substances,Oxidative quinone substances from terrestrial sources
C3	EX325/Em325,420	Humus like substances, Degraded semiquinone substances

Figure S1 The three fluorescence fractions and loading maps of DOM of paddy soil. (A–C): represented the different fluorescence fractions; D: Different Fluorescence characteristics and source analysis

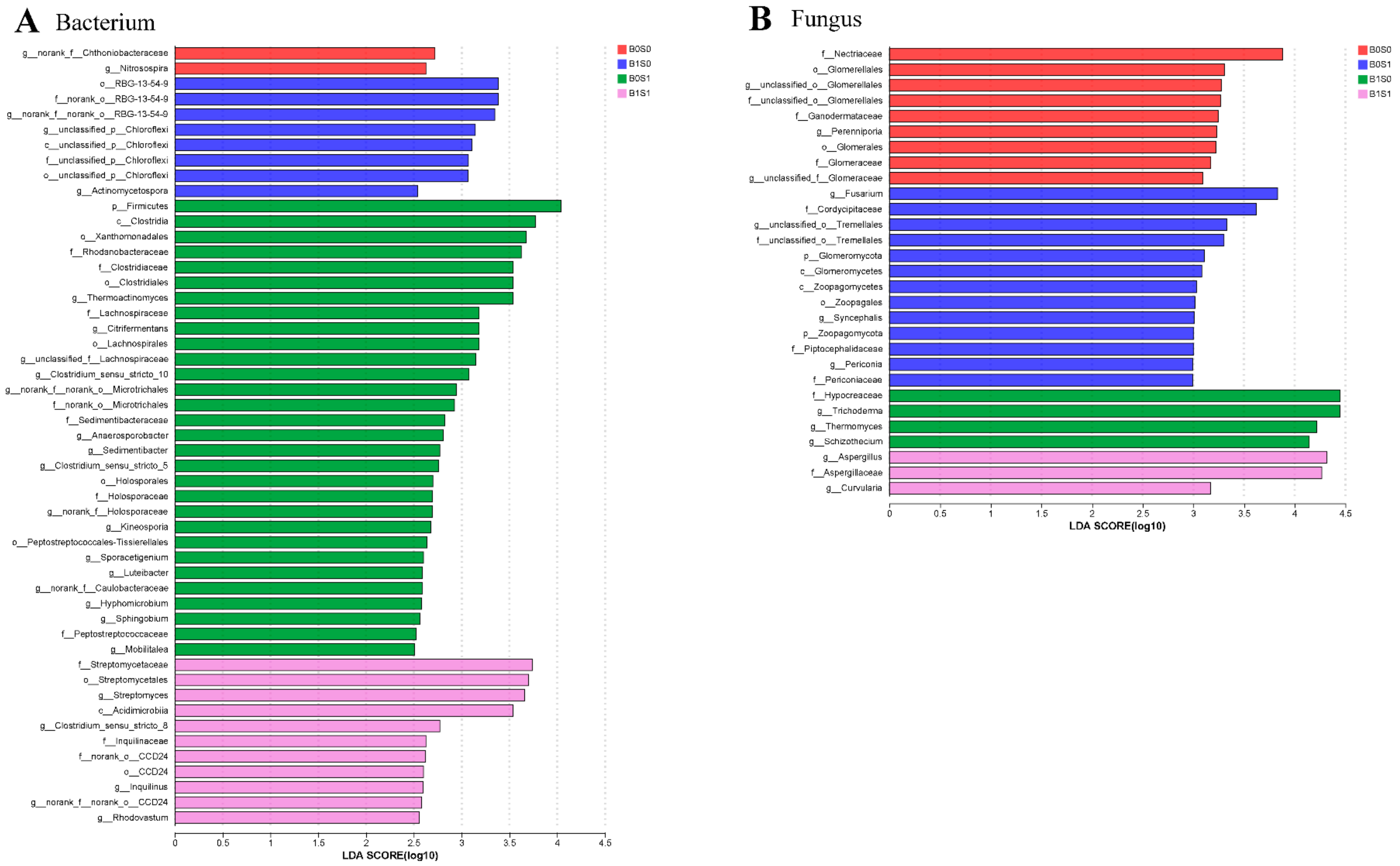

Figure S2 The LEfSe analysis of soil bacterial (A) and fungus (B) under different management measures. The taxa with significantly different abundances among different treatments are represented by dots with different colors, and from the center outward, they represent the phylum, class, order, family, and genus levels.
